# Corrosion-Engineered Morphology and Crystal Structure Regulation toward Fe-Based Efficient Oxygen Evolution Electrodes

**DOI:** 10.3390/nano12121975

**Published:** 2022-06-08

**Authors:** Ying Wang, Zhengbang Yang, Zhonghua Zhang, Ming He

**Affiliations:** 1 State Key Laboratory of Biobased Material and Green Papermaking, Qilu University of Technology (Shandong Academy of Sciences), Jinan 250353, China; y18895787269@163.com; 2 Key Laboratory for Liquid-Solid Structural Evolution and Processing of Materials (Ministry of Education), School of Materials Science and Engineering, Shandong University, Jinan 250061, China; zh_zhang@sdu.edu.cn

**Keywords:** dealloying, corrosion engineer, layered double hydroxides, electrocatalyst, oxygen evolution reaction

## Abstract

The rational regulation of catalysts with a well-controlled morphology and crystal structure has been demonstrated effective for optimizing the electrochemical performance. Herein, corrosion engineering was employed for the straightforward preparation of FeAl layered double hydroxide (LDH) nanosheets and Fe_3_O_4_ nanooctahedrons via the feasible modification of dealloying conditions. The FeAl-LDH nanosheets display an excellent catalytic performance for oxygen evolution reactions in 1 M KOH solution, such as low overpotentials (333 mV on glass carbon electrode and 284 mV on Ni foam at 10 mA cm^−2^), a small Tafel slope (36 mV dec^−1^), and excellent durability (24 h endurance without deactivation). The distinguished catalytic features of the FeAl-LDH nanosheets comes from the Al and Fe synergies, oxygen vacancies, and well-defined two-dimensional (2D) layered LDH structure.

## 1. Introduction

Electrochemical water splitting has received a lot of press as it can not only realize the green transformation from electric energy to chemical energy but also provide a promising platform for intermittent renewable energy utilization (e.g., wind and solar) [1,2,3]. The efficiency-determining process in water splitting is the anodic oxygen evolution reaction (OER), which is kinetically sluggish due to the complicated proton-coupled electron transfer, resulting in a relatively larger overpotential [4,5]. Iridium and ruthenium oxides (RuO_2_ and IrO_2_) represent the state-of-art OER catalysts, but the low reserve and expensive cost impede their practical use [6]. Hence, the development of cost-effective and high-performance OER electrocatalysts is greatly desired.

Recently, transition metals and corresponding derivatives (e.g., chalcogenides [7], carbides [8], nitrides [9], phosphides [10], borides [11], etc.) have come to the foreground in the field of water splitting for their enormous natural resources. Among various candidates, transition metal-based oxides and (oxy)hydroxides are regarded attractive OER electrocatalysts owing to their valence variability and optimal interaction between metal ions and oxygen intermediates [12]. Layered double hydroxides (LDHs) are a type of two-dimensional (2D) layered material that is made up of positively-charged brucite-like host layers coupled with charge-compensating interlayer anions in an alternate pattern [13]. This unusual structure offers a lot of flexibility range for metal species and ratios in the intra layers, providing greater options for customizing the composition and electrical structure of LDHs materials [14,15]. The layered and relatively open structure of LDHs can also accelerate the ion/electron transfer rates, which are of great potential toward efficiently catalyzing OER [16]. Consequently, numerous endeavors have been undertaken to develop various methods for synthesizing the nanostructured metal oxides and LDHs. A series of fabrication routes including the hydrothermal process, coprecipitation, and electrodeposition has been proposed [17,18,19]. However, most of these approaches entail high-temperature processing, the excessive use of organic or capping chemicals, as well as multi-step processes. Therefore, it is crucial to develop a simple and reliable synthetic protocol to fabricate nanostructured metal oxides and LDHs in high throughput.

Dealloying generally refers to the (electro)chemical corrosion process that allows the active elements in the alloy to selectively dissolve into the electrolyte, leaving the non-active elements to form porous microstructures [20]. Over the past decades, dealloying has primarily developed into one of the most common tools for producing useful porous metals and alloys, such as nanoporous gold [21,22]. Less attention has been paid that such facile process could be popularized for the preparation of transition metal-based oxides and (oxy)hydroxides [23]. Herein, dealloying is implemented to prepare FeAl-LDH nanosheets and Fe_3_O_4_ nanooctahedrons based on the elaborate design of the alloy precursor and control of dealloying conditions. A flexible selection over the morphology and crystal structure of the as-dealloyed products could be achieved through corrosion engineering. The FeAl-LDH nanosheets present excellent catalytic activity and stability toward water oxidation in alkaline electrolyte, which is superior to many advanced Fe-based electrocatalysts. Such admirable performance may be assigned to the synergistic effect between Al and Fe with an optimal electronic structure for the enhanced activity, promotion effect of oxygen vacancies on the catalytic activity intrinsically, and well-defined 2D layered LDH nanostructure accelerating the ion/electron diffusion.

## 2. Materials and Methods

### 2.1. Material Synthesis

The Al_98_Fe_2_ precursor alloy ingot was prepared by melting pure metals Al and Fe (purity 99.9 wt. %) in a quartz crucible using a high-frequency induction furnace and then casting the molten metal in a casting mold (Figure S1a). Then, 3~5 g alloy ingots were cut and re-melted into quartz tubes by high-frequency induction heating, and the alloy ribbons were obtained on copper rolls with a diameter of 0.5 m at 1000 rpm using a single-roller rapid cooling device (Figure S1b). The Al_98_Fe_2_ alloy ribbons were immersed in 2 or 5 M NaOH solutions for 6 h at room temperature until no obvious bubbles appeared. After dealloying, the as-obtained powders were repeatedly washed with ultrapure water and collected after drying in a vacuum oven at 60 °C (Figure S1c,d).

### 2.2. Characterizations

The XRD characterizations of precursor ribbon and as-dealloyed samples were performed on a PANalytical’s Empyrean Diffractometer with Cu-Kα radiation (λ = 1.54178 Å) at 40 kV and 30 mA. The morphologies of the as-dealloyed samples were first explored by dispersing on conductive tape using a JEOL JSM-7400F scanning electron microscope (SEM) equipped with an energy-dispersive X-ray (EDX) spectroscopy for analyzing composition. Transmission electron microscopy (TEM), high-resolution TEM (HRTEM), and selected area electron diffraction (SAED) were collected with JEOL JEM-2100F for FeAl-LDH nanosheets and TF 20 analytical TEM for Fe_3_O_4_ nanooctahedrons. In addition, elemental mapping analysis and scanning transmission electron microscopy (STEM) were obtained at a high-angle annular dark-field (HAADF) mode. The chemical structures of as-dealloyed samples were studied by Fourier transform infrared (FTIR/ATR, Bruker ALPHA, Berlin, Germany) with a 4 cm^−1^ spectral resolution. The ESCALAB 250 ultrahigh vacuum (1 × 10^−9^ bar) apparatus was employed for the X-ray photoelectron spectroscopy (XPS) measurements using an Al Kα X-ray source.

### 2.3. Electrochemical Measurements

Electrochemical measurements were conducted in a three-compartment electrochemical glass cell on a Metrohm autolab potentiostat, with a graphite rod as a counter electrode and an Ag/AgCl (saturated KCl) electrode as a reference electrode. The linear sweep voltammetry (LSV) was conducted in 1 M KOH electrolyte at 5 mV s^−1^ under a rotation speed of 1600 rpm at room temperature. The catalysts-modified glass carbon electrode (GCE, 0.196 cm^2^) and Ni foam were prepared as the working electrodes. Catalyst inks were prepared by ultrasonicating the mixture of the catalysts and XC-72 carbon powder (at a mass ration of 4:6), 0.5 mL 0.5 wt% Nafion solution, and 1.5 mL isopropanol for 30 min. Finally, 5 μL catalyst suspension was dropped onto the polished GCEs and then dried in a vacuum tank at ambient temperature overnight (loading: 150 μg cm^−2^). For the catalysts-loaded Ni foam, the ink was prepared by dispersing FeAl-LDH nanosheets in ethanol in a 4 mL glass vial with a 2 mg mL^−1^ concentration; then, 5 *v*/*v* % of Nafion was added. The inks were sonicated for 30 min for fully mixing. Then, 8 μL of the ink was drop-casted onto 1 cm × 1 cm Ni foam, resulting in 16 μg of catalyst loading. Prior to the electrochemical measurements, oxygen gas was bubbled into 1 M KOH electrolyte for 30 min. The electrochemically active surface areas (ECSAs) were evaluated from the electrochemical double-layer capacitance (C_dl_), which was collected by recording cyclic voltammograms values at different scan rates (10, 20, 30, 40, and 50 mV·s^−1^) in the non-faradaic path range. EIS measurements were performed in 1 M KOH electrolyte at an excitation voltage of 5 mV with frequencies ranging from 10^−2^ to 105 Hz. All polarization curves were corrected with 100% iR compensation. The Nernst equation (ERHE = EAg/AgCl + 0.198 V + 0.059 pH) was adopted to convert the potentials to reversible hydrogen electrode (RHE). Long-term durability was revealed by the chronopotentiometry test at a constant current density of 10 mA cm^−2^, while the LSV curves before and after durability tests were also recorded for comparison.

## 3. Results and Discussion

### 3.1. Preparation of FeAl-LDH Nanosheets and Fe_3_O_4_ Nanooctahedrons

**Figure 1:**
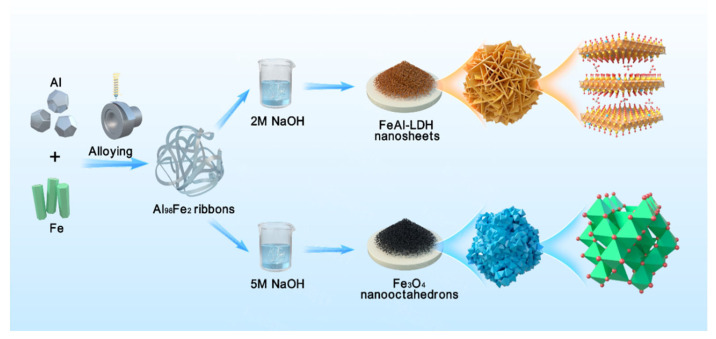
Schematic illustration showing the corrosion-engineered fabrication process of the FeAl-LDH nanosheets and Fe_3_O_4_ nanooctahedrons.

Al-based alloy is generally preferred as the dealloying precursor because the selective sacrifice of Al in NaOH solution is conducive to the surface diffusion and reconstruction of the nanostructure [24]. Iron corrosion is a common phenomenon during which iron always spontaneously forms an oxidized structure in contact with air and water [25]. The slight richness of Al in the precursor satisfies the common criteria of the dealloying process. Therefore, the Al_98_Fe_2_ alloy was meticulously designed as a precursor. Figure 1 illustrates the corrosion-engineered process for the Al_98_Fe_2_ precursor, wherein different NaOH concentrations were adopted to regulate the crystal structures and morphologies of the as-dealloyed samples.

### 3.2. Characteristics of FeAl-LDH Nanosheets and Fe_3_O_4_ Nanooctahedrons

#### 3.2.1. The XRD Analysis of FeAl-LDH Nanosheets and Fe_3_O_4_ Nanooctahedrons

**Figure 2:**
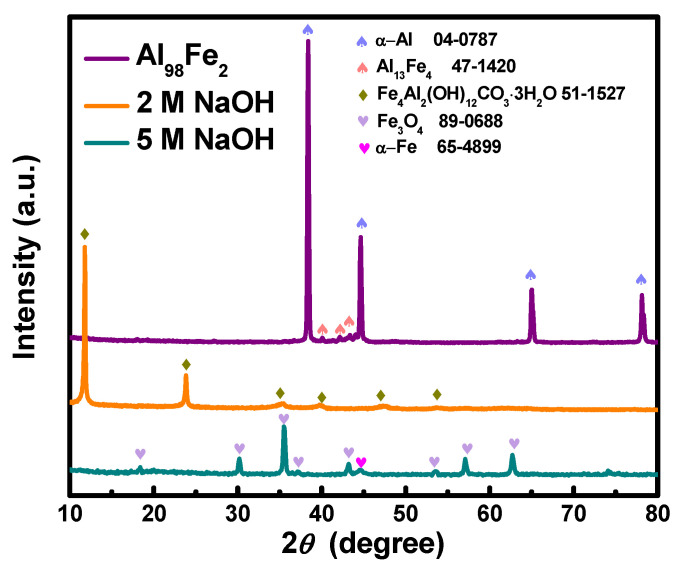
Typical XRD patterns of Al_98_Fe_2_ precursor ribbons and the two as-dealloyed samples.

According to the XRD results (Figure 2), the rapidly solidified Al_98_Fe_2_ ribbon consists of the Al phase (JCPDS No. 04-0787) and the Al_13_Fe_4_ intermetallic phases (JCPDS No. 47-1420). After dealloying in 2 M NaOH, the XRD pattern corresponds to the Fe_4_Al_2_(OH)_12_CO_3_·3H_2_O phase (JCPDS No. 51-1527), with three landmark diffraction peaks at 11.6°, 23.4°, and 34.1°, reflecting the (003), (006), and (222) planes of the well-known binary LDH structure. The position of the (003) peak is derived from the interlayer separation induced by the occupation of CO_3_^2−^ ions and water molecules [16]. In contrast, the XRD pattern obtained after dealloying in 5 M NaOH is mainly in the Fe_3_O_4_ phase (JCPDS No. 89-0688), with a small peak for the α-Fe phase (JCPDS No. 65-4899).

#### 3.2.2. The Morphologies of FeAl-LDH Nanosheets and Fe_3_O_4_ Nanooctahedrons

**Figure 3:**
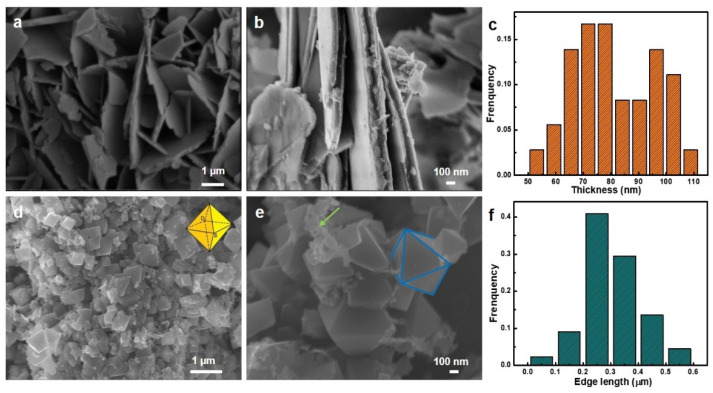
SEM images showing the morphology of products obtained by the dealloying Al_98_Fe_2_ ribbons in the (**a**,**b**) 2 and (**d**,**e**) 5 M NaOH solutions. The columnar plots showing the (**c**) thickness distribution of the FeAl-LDH nanosheets and the (**f**) edge length distribution of Fe_3_O_4_ nanooctahedrons, respectively.

The morphologies of the two as-dealloyed samples were first revealed by SEM. As shown in Figure 3a,b and Figure S2a, the as-dealloyed sample obtained in 2 M NaOH presented an irregular nanosheets structure, and the thickness of nanosheets was found to be ca. 85 nm (Figure 3c), whereas regular Fe_3_O_4_ nanooctahedrons with an average edge length of 300 nm were dominated after dealloying in 5 M NaOH (Figure 3d–f and Figure S3a). Some small nanoparticle clusters (shown by the green arrows) could also be detected, which were attributed to the α-Fe phase. In addition, the elemental compositions of the two as-dealloyed samples were analyzed by SEM-EDX (Figure S2b and Figure S3b), and the lower Al content of the as-dealloyed sample in 5 M NaOH was due to the accelerated Al corrosion rate by the high OH- concentration. For convenience, the two as-dealloyed samples are named FeAl-LDH NSs and Fe_3_O_4_ NOs, respectively.

**Figure 4:**
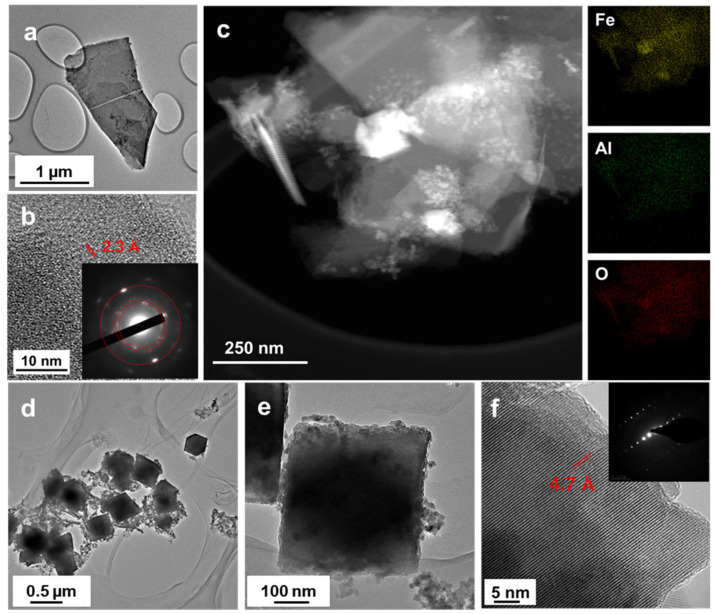
(**a**) TEM, (**b**) HRTEM, (**c**) STEM, and corresponding elemental mapping images of FeAl-LDH nanosheets. Inset (**b**) corresponding SAED pattern. (**d**,**e**) TEM and (**f**) HRTEM images of Fe_3_O_4_ nanooctahedrons. Inset (**f**) corresponding SAED pattern.

TEM image of FeAl-LDH NSs confirms the nanosheets morphology again (Figure 4a). Further examination by HRTEM exposes the (225) plane of the LDH with a lattice spacing of 2.3 Å (Figure 4b). The SAED pattern reflects the nanocrystalline nature of the FeAl-LDH NSs, and the diffraction rings matched well with the (222), (225), and (600) planes of FeAl-LDH (inset of Figure 4b). The HAADF-STEM image of FeAl-LDH nanosheet coupled with the corresponding EDX elemental mapping analysis reveals the uniform distribution of Fe, Al, and O elements throughout all the nanosheets (Figure 4c). Figure 4d and Figure S4 exhibit the TEM images of the Fe_3_O_4_ octahedra particles, in which diamond projection and square projection can be visualized. The clear contrast of brightness between the edge and the center verifies the octahedron structure of particles (Figure 4e). The HRTEM image shows the lattice stripes covering the entire structure of the particle, implying that the Fe_3_O_4_ nanooctahedrons have a single-crystal nature (Figure 4f). The interfacial distance is measured to be about 4.7 Å, which is related to the (111) plane. In addition, the SAED pattern (inset Figure 4f) further elucidate so a monocrystalline structure recorded along the (111) zone axis direction.

#### 3.2.3. FTIR Spectra of FeAl-LDH Nanosheets and Fe_3_O_4_ Nanooctahedrons and XPS Spectra for Feal-Ldh Nanosheets

**Figure 5:**
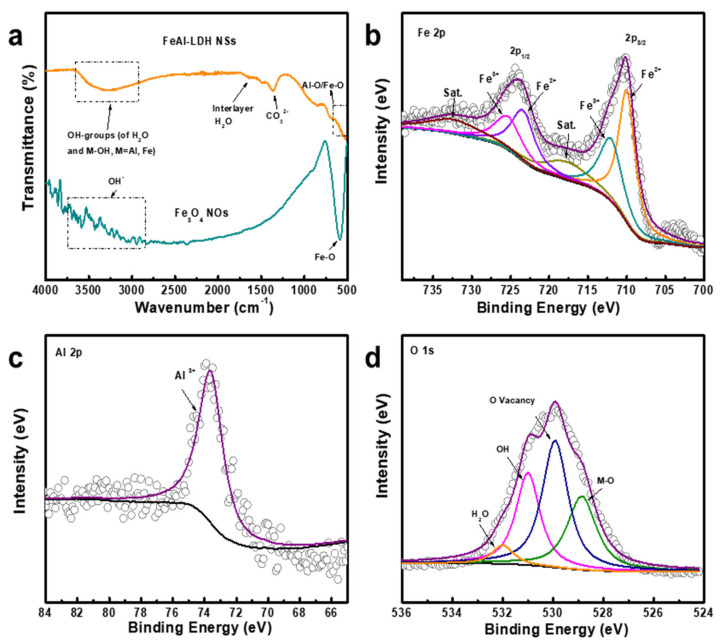
(**a**) FTIR spectra of FeAl-LDH nanosheets and Fe_3_O_4_ nanooctahedrons. (**b**) Fe 2p, (**c**) Al 2p, and (**d**) O 1s XPS spectra for FeAl-LDH nanosheets.

FTIR spectroscopy was employed to analyze the function groups and chemical structures of the FeAl-LDH NSs and Fe_3_O_4_ NOs (Figure 5a). As for FeAl-LDH NSs, the broad absorption band in the range of 2900–3600 cm^−1^ corresponds to the stretching vibrations of OH groups in the intralayers (M–OH, M = Fe and Al) and water molecules in the interlayer [26]. The bending pattern for H_2_O in the interlayer appears at 1634 cm^−1^, implying the existence of water molecules as bending vibrations [27]. The sharp absorption peak at 1362 cm^−1^ is related to the O–C–O unidentate carbonate symmetric stretching vibrations of CO_3_^2−^ in the interlayer [28]. Some weak bands at the low frequency region of 800–500 cm^−1^ reflect the vibration of metal oxygen bonds for the brucite-like lattice characteristic of layered solids [29]. The FTIR spectrum of Fe_3_O_4_ NOs shows a strong peak of 580 cm^−1^, a typical characteristic for the vibrations of the Fe–O bonds in magnetite [30]. It is worth noting that a number of adsorption bands in the range of 2800–4000 cm^−1^ originated from the isolated hydroxylgroups or O–H stretching vibration in H_2_O, revealing that the OH^−^ is adsorbed on the Fe_3_O_4_ surface [31].

The surface chemical states of FeAl-LDH NSs and Fe_3_O_4_ NOs were further elucidated by XPS (Figure 5b–d and Figure S5). In Figure 5b, the Fe 2p spectrum of FeAl-LDH NSs can be fitted into two pairs of doublets. The major one (at 710.0 and 723.6 eV) and the minor one (at 712.2 and 725.6 eV) can be assigned to the Fe^2+^ and Fe^3+^ state [32], respectively, and two satellites can also be detected. The Al 2p XPS peak located at 73.7 eV is attributed to Al^3+^, corresponding to the aluminum oxide (Al_2_O_3_) (Figure 5c) [33]. The binding energy of Al 2p peak shifts compared to the standard value (74.7 eV), indicating that the transfer of electrons occurs between Al and Fe [34]. Moreover, the fitted O 1s spectrum shows four peaks located at approximately 528.9 eV, 529.9 eV, 531.1 eV, and 532.0 eV, corresponding to the metal–oxygen (Fe–O/Al–O) bond, oxygen vacancy, metal hydroxyl group (Fe-OH/Al–OH), and absorbed water, respectively (Figure 5d) [35]. As for Fe_3_O_4_ NOs, the peak deconvolution of Fe 2p spectrum also proves the coexistence of Fe^2+^ (710.0 and 723.6 eV) and Fe^3+^ (712.1 and 725.7 eV) (Figure S5b) [36]. The O 1s XPS spectrum of Fe_3_O_4_ NOs may be divided into four peaks: 539.3 eV for Fe–O, 530.0 eV for oxygen vacancy, and 531.0 eV for the hydroxyl group (Figure S5c) [37].

Based on the above characterization results, the structure and morphology evolution under different NaOH concentrations can be addressed as follows. When treated in 2 M NaOH solution, Al_98_Fe_2_ ribbons first collide with the OH^−^ ions forming the intermediate Fe(OH)_2_ phase [38]. The whole dealloying process is not too intense due to the relatively low OH^−^ concentration, which provides enough time for Fe(OH)_2_ contacting with CO_2_ in the air, thereby introducing carbonate combined with retaining the partial Al forming Fe_4_Al_2_(OH)_12_CO_3_·3H_2_O-layered double hydroxide [16]. Additionally, the 2D nanosheets structure is formed by stacking the laminates with the positive charge of metal ions. Upon leaching Al atoms in 5 M NaOH solution, the Fe atoms will oxidize rapidly to form an Fe_3_O_4_ crystal nucleus under the influence of a large amount of released reaction heat. The subsequent nucleation growth prefers adopting a cubic structure to minimize the total surface free energy. Meanwhile, the surface energies follows the order of γ (111) < γ (100) < γ (110) [39], resulting in a low energy form of Fe_3_O_4_ octahedron surrounded by eight (111) planes. The previous hydrothermal synthesis for iron oxides also indicates an increase in the alkalinity favors of the formation of Fe_3_O_4_ octahedra [40]. This formation mechanism can be supported by our FTIR results that OH^−^ strongly adsorbed on the Fe_3_O_4_ (111) surface [41].

### 3.3. The Electrochemical Measurements s of the FeAl-LDH NSs and Fe_3_O_4_ Nos

**Figure 6:**
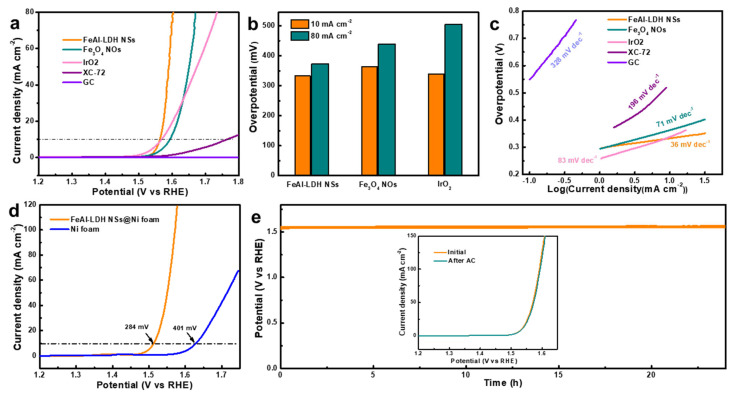
(**a**) OER polarization curves, (**b**) overpotentials at 10 and 80 mA cm^−2^, and (**c**) the related Tafel slopes of different catalysts loaded on GCE at 5 mV s^−1^ in 1 M KOH solution. (**d**) OER polarization curves of FeAl-LDH NSs loaded on Ni foam and barely Ni foam at 5 mV s^−1^ in 1 M KOH solution. (**e**) Long-term stability results of the FeAl-LDH NSs@Ni foam electrode with a constant current density of 10 mA cm^−2^ with inset (**e**) polarization curves of FeAl-LDH NSs@Ni foam before and after the stability measurement.

The electrocatalytic OER activities of the as-obtained FeAl-LDH NSs and Fe_3_O_4_ NOs were first evaluated by a rotating disk electrode in 1.0 M KOH. Similar tests were carried out on commercial IrO_2_, XC-72 carbon powder, and blank GCE for comparison. Figure 6a plots the *iR*-corrected polarization curves of OER at 5 mV s^−1^. It is obvious that XC-72 carbon exhibits negligible OER activity, while GCE scarcely possesses activity. To launch a current density of 10 mA cm^−2^, overpotentials of 333, 364, and 339 mV are required for FeAl-LDH NSs, Fe_3_O_4_ NOs, and commercial IrO_2_, respectively. To obtain a higher current density of 80 mA cm^−2^, the FeAl-LDH NSs display a much lower overpotential of 373 mV compared to those of the Fe_3_O_4_ NOs (440 mV) and commercial IrO_2_ (505 mV) (Figure 6b). The rapidly increasing current density of FeAl-LDH NSs during the OER process indicates its excellent reaction kinetics. The Tafel plots derived from the polarization curve were further examined to acquire more information about the OER kinetics (Figure 6c). Remarkably, the FeAl-LDH NSs catalyst has the lowest Tafel slope of 36 mV dec^−^^1^, in contrast to the 71 mV dec^−1^ of Fe_3_O_4_ NOs and 83 mV dec^−1^ of commercial IrO_2_, revealing the admirable OER electrocatalytic kinetic and activity of FeAl-LDH NSs. The Tafel slope can also provide vital information about the rate determination step (RDS). Generally, a Tafel slope would be 120 mV dec^−1^ if the first step of the OER with one electron transfer is the RDS, a Tafel slope of 60 mV dec^−1^ if the second step of the OER with two electron transfers is the RDS, and a Tafel slope of 40 mV dec^−1^ if the third step of the OER with three electron transfers is the RDS [42]. Hence, the rate determination step for FeAl-LDH NSs corresponds to the third step. EIS could further disclose the underlying electrochemical behavior at the catalyst/electrolyte interface. Figure S6 shows the equivalent circuit in which *R*_ct_, *R*_s_, and CPE represent the charge transfer resistance, electrolyte resistance, and constant phase element, respectively. The charge transfer resistance (*R*_ct_), i.e., diameter of the semi-circle, is an important parameter of electrochemical reaction activity. The fitted *R*_ct_ values of FeAl-LDH NSs, Fe_3_O_4_ NOs, and IrO_2_ are 4206, 6572, and 19,133 kΩ, respectively, and the smallest *R*_ct_ of FeAl-LDH NSs implies the fastest charge transfer kinetic during electrocatalysis. It is worth mentioning that Supplementary Table S1 includes a comparison with other previously published Fe-based OER electrocatalysts, demonstrating that our as-prepared FeAl-LDH NSs catalyst has exceptional an OER performance that is comparable to or better than other Fe-based OER electrocatalysts. The electrochemical surface areas (ECSA) of FeAl-LDH NSs and Fe_3_O_4_ NOs were analyzed from their electrochemical double-layer capacitances (C_dl_) by cyclic voltammetry with different scan rates in the non-faradaic path range. Figure S7 demonstrates the CV curves of FeAl-LDH NSs and Fe_3_O_4_ NOs, and the corresponding C_dl_ values for FeAl-LDH NSs and Fe3O4 NOs are 2.5 and 1.4 mF cm^−2^, respectively. The higher C_dl_ value of FeAl-LDH NSs indicates expose more active sites for electrocatalysis Ni foam, as an electrocatalyst substrate is valued for its inexpensive cost and high electrical conductivity, as well as being more suitable to a supported catalyst for long-term service than GCE. Herein, FeAl-LDH NSs were also deposited on Ni foam to further study the OER electrocatalytic performance in 1 M KOH (Figure 6d and Figure S8). Obviously, a notable enhancement of the Ni foam-supported FeAl-LDH NSs electrode for current response to OER can be observed, with an ultralow overpotential of 284 mV at a current density of 10 mA cm^−2^. In comparison, the OER activity of Ni foam is quite low, reflecting the excellent catalytic activity of FeAl-LDH NSs. In addition, durability is another key parameter for determining the suitability of an electrocatalyst for commercial use. Figure 6e shows the chronoamperometry curve of an FeAl-LDH NSs@Ni foam electrode measured at a constant current density of 10 mA cm^−2^. The electrode displayed changeless overpotential during the 24 h durability test, while the LSV curves before and after chronopotentiometric measurement were almost coincident, indicating the excellent OER catalytic stability (inset Figure 6e).

In view of the above-mentioned results, FeAl-LDH nanosheets exhibit superior OER activity and durability and thus might serve as a new potential OER electrocatalyst. The excellent OER performance of FeAl-LDH nanosheets may be associated with the following effects. (i) The synergistic impact between Al and Fe favoring the electronic structure modifies the interaction intensity of the involved adsorbates to the catalyst surface, potentially facilitating reaction kinetics. (ii) The oxygen vacancies providing large numbers of low coordination defects could improve the electrophilicity of the adsorbed O and promote the OH adsorption over the active sites. (iii) The well-defined 2D layered LDH nanostructure can significantly expedite the transfer for molecules, ions, and electrons.

## 4. Conclusions

In summary, FeAl-LDH nanosheets and Fe_3_O_4_ nanooctahedrons were fabricated via simply combining a rapid solidification technique with subsequent different dealloying processes. The corrosion-engineered mechanism of regulating the morphology and crystal structure was bound up with the concentration of the NaOH solution. Furthermore, FeAl-LDH nanosheets show superior OER activity and durability in an alkaline solution. The unique 2D nanosheets morphology, the synergy of Al and Fe, existence of oxygen vacancies, as well as the accelerated charge/ion transfer of LDH structure account for the well-promoted reaction kinetic and electrocatalytic performance. This work not only provides a bright way to prepare a low-cost electrocatalyst toward water oxidation but also a practical guide to layout various nanostructured materials using corrosion science.

## Data Availability

Not applicable.

## Supplementary Materials

The following supporting information can be downloaded at: https://www.mdpi.com/article/10.3390/nano12121975/s1, Figure S1: Photographs of (a) Al_98_Fe_2_ alloy ingot, (b) Al_98_Fe_2_ alloy ribbons, and as-obtained powders after dealloying in (c) 2 or (d) 5 M NaOH solutions; Figure S2: (a) SEM image and (b) typical EDX spectrum of the FeAl-LDH nanosheets. The corresponding compositions are listed in Figure S2b; Figure S3: (a) SEM image and (b) typical EDX spectrum of the Fe_3_O_4_ nanooctahedrons. The corresponding compositions are listed in Figure S3b; Figure S4: TEM images of Fe_3_O_4_ nanooctahedrons; Figure S5: (a) XPS survey spectra of FeAl-LDH nanosheets and Fe_3_O_4_ nanooctahedrons (b) Fe 2p and (c) O 1s spectra of Fe_3_O_4_ nanooctahedrons; Figure S6: Nyquist diagrams of the catalysts loaded on GCE in 1 M KOH; Figure S7. CVs and linear fit of the capacitive current vs scan rates for (a,b) FeAl-LDH NSs and (c,d) Fe_3_O_4_ NOs at scan rates of 5, 10, 20, 30, 40, and 50 mV s^−1^; Figure S8. (a) Tafel slopes and (b) Nyquist diagrams of the FeAl-LDH NSs@Ni foam elctrode and barely Ni foam electrode in 1 M KOH; Table S1. Comparisons of OER activities of FeAl-LDH nanosheets with those of recently reported Fe-basesd OER catalysts. References [43,44,45,46,47,48,49,50,51,52] are cited in the supplementary materials.
