# Supplementary material for: Corrosion-Engineered Morphology and Crystal Structure Regulation toward Fe-Based Efficient Oxygen Evolution Electrodes
Source: Nanomaterials (Basel). 2022 Jun 8;12(12):1975. doi: 10.3390/nano12121975 (PMC9228532; doi:10.3390/nano12121975)
Supplement: Supplementary file 1 [file nanomaterials-12-01975-s001.zip › nanomaterials-1751028-supplementary.pdf]

nanomaterials

Supplementary Materials

Corrosion-Engineered Morphology and Crystal Structure Regulation toward Fe-Based Efficient Oxygen Evolution Electrodes

Ying Wang [1,*,†], Zhengbang Yang [1,†], Zhonghua Zhang [2] and Ming He [1,*]

[1] State Key Laboratory of Biobased Material and Green Papermaking, Qilu University of Technology (Shandong Academy of Sciences), Jinan 250353, China; y18895787269@163.com
[2] Key Laboratory for Liquid-Solid Structural Evolution and Processing of Materials (Ministry of Education), School of Materials Science and Engineering, Shandong University, Jinan 250061, China; zh_zhang@sdu.edu.cn
* Correspondence: wangying93@qlu.edu.cn (Y.W.); heming8916@qlu.edu.cn (M.H.)
† These authors have contributed equally to this work.

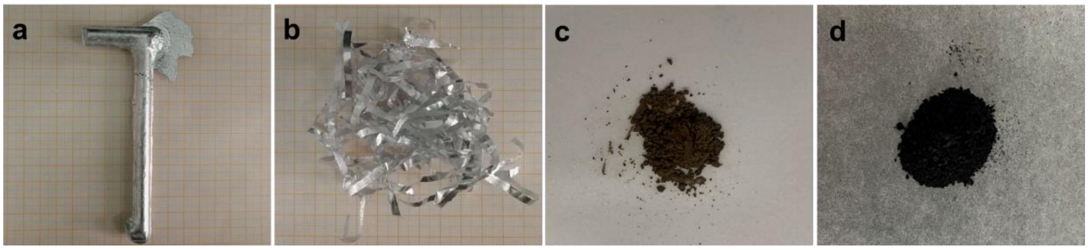

Figure S1. Photographs of (**a**) $Al_{98}Fe_2$ alloy ingot, (**b**) $Al_{98}Fe_2$ alloy ribbons, and as-obtained powders after dealloying in (**c**) 2 or (**d**) 5 M NaOH solutions.

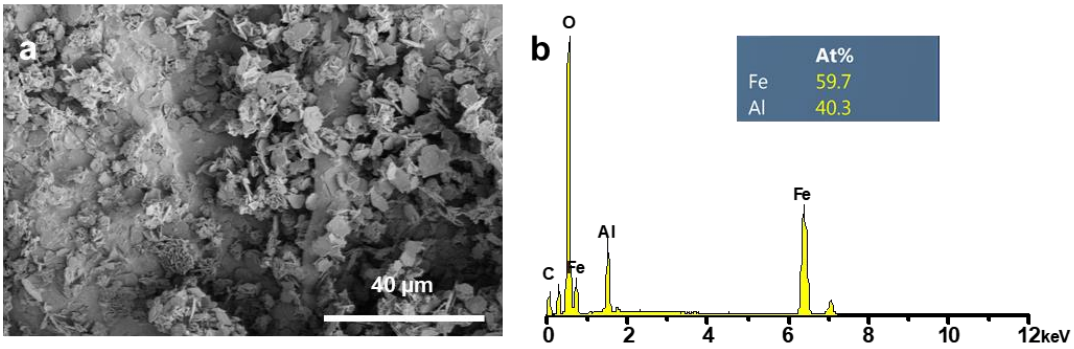

Figure S2. (**a**) SEM image and (**b**) typical EDX spectrum of the FeAl-LDH nanosheets. The corresponding compositions are listed in Figure S2b.

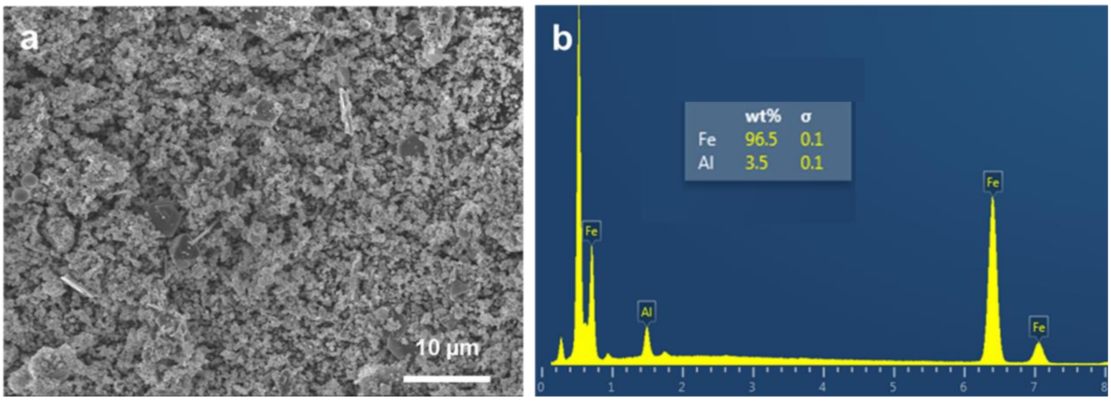

Figure S3. (**a**) SEM image and (**b**) typical EDX spectrum of the Fe_3O_4 nanooctahedrons. The corresponding compositions are listed in Figure S3b.

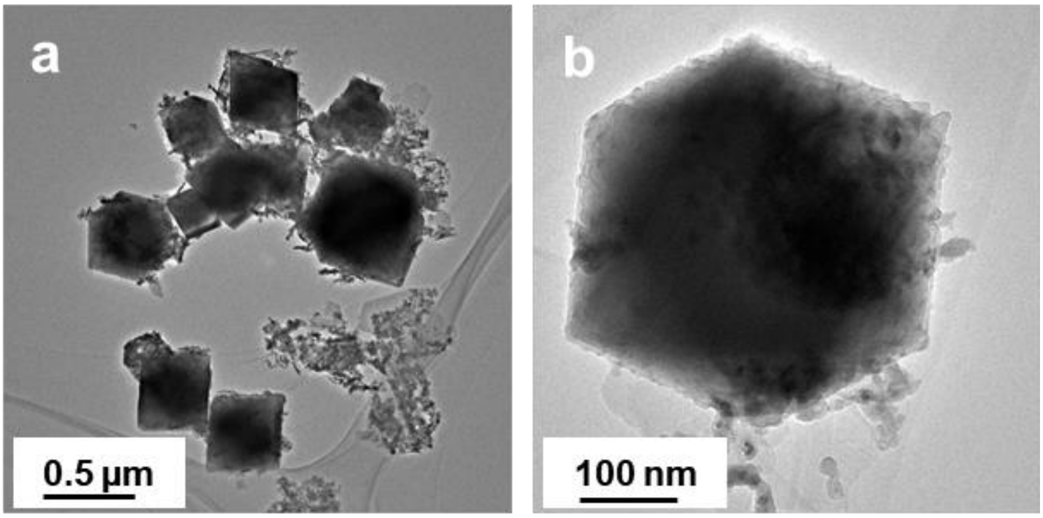

Figure S4. TEM images of Fe_3O_4 nanooctahedrons.

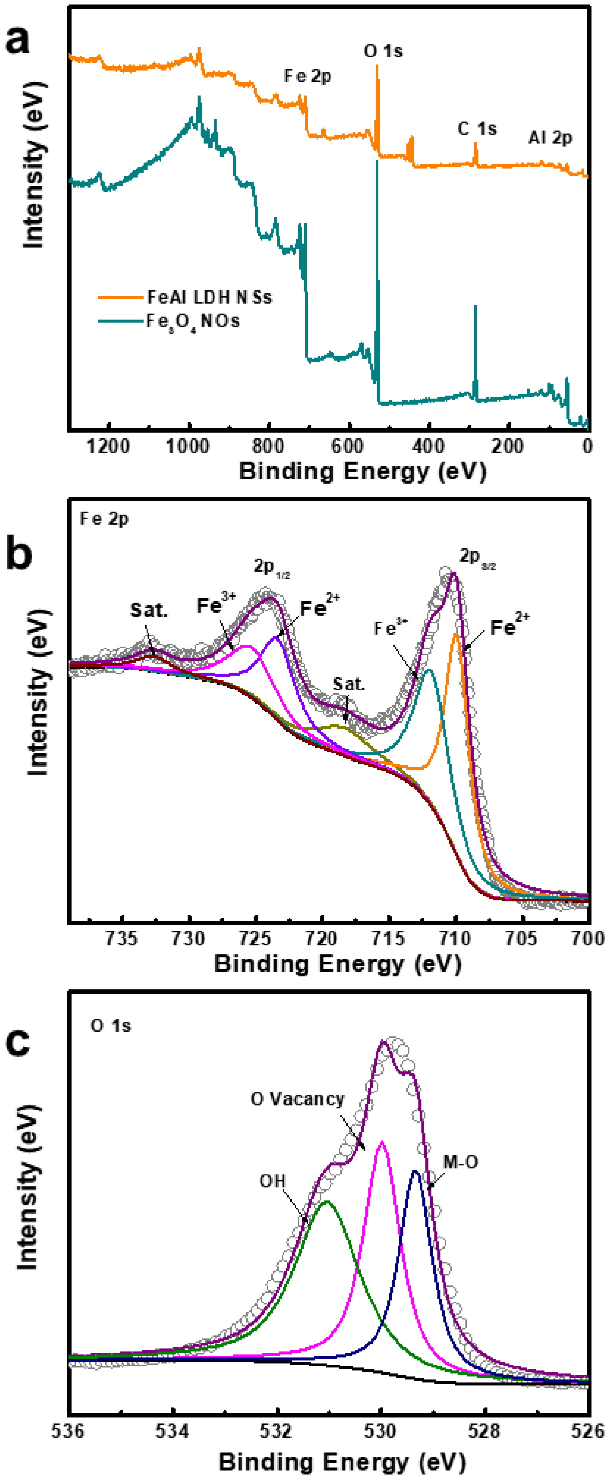

Figure S5. (**a**) XPS survey spectra of FeAl-LDH nanosheets and Fe_3O_4 nanooctahedrons (**b**) Fe 2p and (**c**) O 1s spectra of Fe_3O_4 nanooctahedrons.

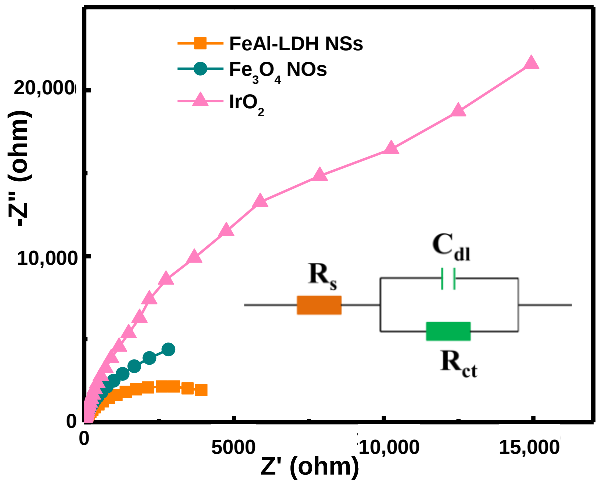

Figure S6. Nyquist diagrams of the catalysts loaded on GCE in 1 M KOH.

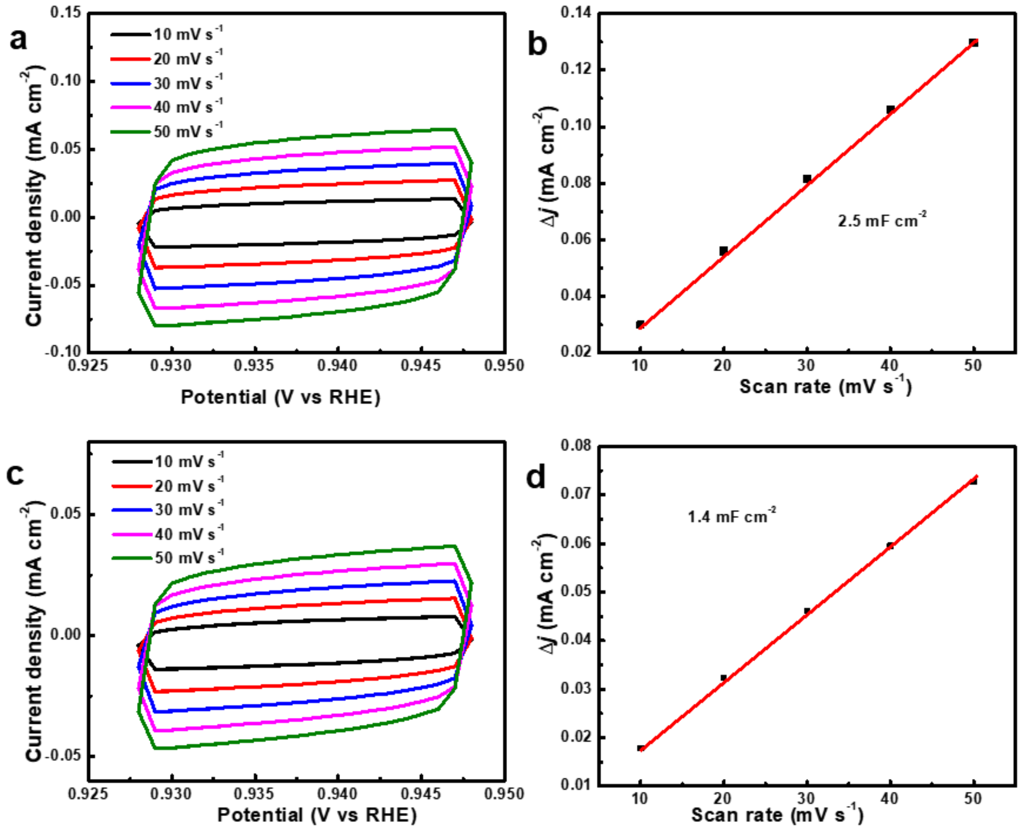

Figure S7. CVs and linear fit of the capacitive current vs scan rates for (**a**,**b**) FeAl-LDH NSs and (**c**,**d**) Fe_3O_4 NOs at scan rates of 5, 10, 20, 30, 40, and 50 mV s^{-1}.

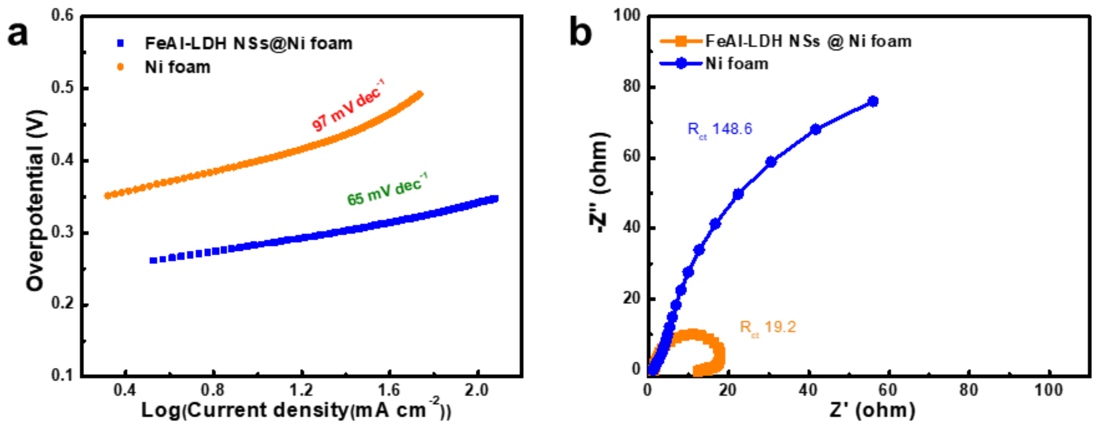

Figure S8. (**a**) Tafel slopes and (**b**) Nyquist diagrams of the FeAl-LDH NSs@Ni foam elctrode and barely Ni foam electrode in 1 M KOH.

Table S1. Comparisons of OER activities of FeAl-LDH nanosheets with those of recently reported Fe-basesd OER catalysts.

Catalyst	Electrolyte	η_{10} (mV)	Tafel (mV dec^{-1})	Refs.
FeAl-LDH nanosheets	1.0 M KOH	**333**	**36**	**This work**
(Fe,Co)-SA/CS	0.1 M KOH	360	109.6	[1]
$LaCo_{0.1}F_{0.9}O_3$	1.0 M KOH	452	N/A	[2]
$MnFe_2O_4$/Ni foam	1.0 M KOH	310	65	[3]
Fe/Fe_3C-N-CNT	1.0 M KOH	340	78	[4]
Fe–36 at% Mn	0.1 M KOH	510	88	[5]
CPF/$FeCoO_x$-Nanoparticles	0.1 M KOH	400	28	[6]
Ni–Fe NPs/Fe	1.0 M KOH	319	41.2	[7]
Fe-CoxP	1.0 M KOH	300	49	[8]
$Fe_3C/CoFe_2O_4$@CNFs-1.5	1.0 M KOH	340	128.4	[9]
CoFe LDHs/Ni foam	1.0 M KOH	300	53	[10]

References

1. Jose, V.; Hu, H.; Edison, E.; Manalastas Jr, W.; Ren, H.; Kidkhunthod, P.; Sreejith, S.; Jayakumar, A.; Nsanzimana, J. M. V.; Srinivasan, M. et al. Modulation of Single Atomic Co and Fe Sites on Hollow Carbon Nanospheres as Oxygen Electrodes for Rechargeable Zn–Air Batteries. *Small Methods* **2021**, *5*, 2000751.
2. Füngerlings, A.; Koul, A.; Dreyer, M.; Rabe, A.; Morales, D. M.; Schuhmann, W.; Behrens, M.; Pentcheva, R. Synergistic Effects of Co and Fe on the Oxygen Evolution Reaction Activity of $LaCo_xFe_{1-x}O_3$. *Chem.-Eur. J.* **2021**, *27*, 17145–17158.
3. Kim, J.; Lee, J.; Liu, C.; Pandey, S.; Woo Joo, S.; Son, N.; Kang, M. Achieving a long-term stability by self-redox property between Fe and Mn ions in the iron-manganese spinel structured electrode in oxygen evolution reaction. *Appl. Surf. Sci*. **2021**, *546*, 149124.
4. Zong, L.; Chen, X.; Dou, S.; Fan, K.; Wang, Z.; Zhang, W.; Du, Y.; Xu, J.; Jia, X.; Zhang, Q. et al**.** Stable confinement of Fe/Fe_3C in Fe, N-codoped carbon nanotube towards robust zinc-air batteries. *Chinese Chem. Lett.* **2021**, *32*, 1121–1126.
5. Konno, Y.; Yamamoto, T.; Nagayama, T. Nanoporous manganese ferrite films by anodising electroplated Fe–Mn alloys for bifunctional oxygen electrodes. *Nanoscale*, **2021**, *13*, 12738–12749.
6. Sha, Q.; Wang, J.; Lu, Y.; Zhao, Z. Polyaniline@MOF fiber derived Fe–Co oxide-based high performance electrocatalyst. *New J. Chem*. **2021**, *45*, 282–287.
7. Xue, R.; Guo, M.; Wei, Z.; Zhang, Q. Deep eutectic solvent-induced synthesis of Ni–Fe catalyst with excellent mass activity and stability for water oxidation. *Green Energy Environ*. **2021**, *in press*. https://doi.org/10.1016/j.gee.2021.11.006.
8. Song, W.; Teng, X.; Niu, Y.; Gong, S.; He, X.; Chen, Z. Self-templating construction of hollow Fe-Co_xP nanospheres for oxygen evolution reaction. *Chem. Eng. J.* **2021**, *409*, 128227.
9. Sun, Y.; Li, Y.; You, S.; Li, X.; Zhang, Y.; Cai, Z.; Liu, M.; Ren, N.; Zou, J. $Fe_3C/CoFe_2O_4$ nanoparticles wrapped in one-dimensional MIL-53(Fe)-derived carbon nanofibers as efficient dual-function oxygen catalysts. *Chem. Eng. J***2021**, *424*, 130460.
10. Liu, R.; Wang, Y.; Liu, D.; Zou, Y.; Wang, S. Water-Plasma-Enabled Exfoliation of Ultrathin Layered Double Hydroxide Nanosheets with Multivacancies for Water Oxidation. *Adv. Mater.* **2017**, 29, 1701546.
